# Isolated Intracranial Hypertensions as Onset of Myelin Oligodendrocyte Glycoprotein Antibody Disease

**DOI:** 10.3390/jcm13154468

**Published:** 2024-07-30

**Authors:** Laura Papetti, Giulia Moltoni, Daniela Longo, Gabriele Monte, Francesco Dellepiane, Stefano Pro, Giorgia Bracaglia, Claudia Ruscitto, Alberto Verrotti, Massimiliano Valeriani

**Affiliations:** 1 Developmental Neurology Unit, Bambino Gesù Children’s Hospital IRCCS, 00165 Rome, Italy; gabriele.monte@opbg.net (G.M.); stefano.pro@opbg.net (S.P.); claudia.ruscitto@opbg.net (C.R.); massimiliano.valeriani@opbg.net (M.V.); 2 Functional and Interventional Neuroradiology Unit, Bambino Gesù Children’s Hospital IRCCS, 00165 Rome, Italydaniela.longo@opbg.net (D.L.); francesco.dellepiane@opbg.net (F.D.); 3 Neuroradiology Unit, NESMOS Department Sant’Andrea Hospital, La Sapienza University, Via di Grottarossa, 1035-1039, 00189 Rome, Italy; 4 Department of Diagnostics and Laboratory Medicine, Medical Laboratory Unit, Unit of Allergy and Autoimmunity, Bambino Gesù Children’s Hospital, IRCCS, 00165 Rome, Italy; giorgia.bracaglia@opbg.net; 5 Department of Pediatrics, University of Perugia, 06123 Perugia, Italy; alberto.verrotti@unipg.it; 6 Systems Medicine Department, Hospital of Rome, Tor Vergata University, 00133 Rome, Italy; 7 Center for Sensory Motor Interaction, Aalborg University, DK-9220 Aalborg, Denmark

**Keywords:** idiopathic intracranial hypertension, pseudotumor cerebri, secondary headache, MOGAD, MOG antibodies

## Abstract

Myelin oligodendrocyte glycoprotein antibody disease (MOGAD) is characterized by multiple phenotypic conditions such as acute disseminated encephalomyelitis, optic neuritis, and myelitis. MOGAD’s spectrum is expanding, with potential symptoms of increased intracranial pressure that are similar to idiopathic intracranial hypertension (IIH). We report a boy with new-onset continuous headache and a brain MRI at onset suggesting idiopathic intracranial hypertension (IIH). The patient showed resistance to treatment with acetazolamide and, after one month, developed optic neuritis in the left eye. Laboratory tests documented positive MOG antibodies (anti-MOG) in the serum. The final diagnosis was MOGAD, with the initial symptoms resembling IIH.

## 1. Introduction

Myelin oligodendrocyte glycoprotein antibody disease (MOGAD) is an autoimmune inflammatory demyelinating disease characterized by the presence of antibodies against glial glycoproteins on the myelin sheath (anti-MOG) [1]. Optic neuritis, transverse myelitis, and encephalomyelitis are possible symptoms depending on the involved areas of the nervous system [2]. In children, the onset of MOGAD mostly occurs with acute disseminated encephalomyelitis (ADEM), whereas in adulthood it occurs with optic neuritis [3]. According to the “International MOGAD panel”, the diagnosis is based on the presence of anti-MOG in the blood, along with clinical and imaging evidence that excludes other demyelinating diseases such as multiple sclerosis [1]. Live cell-based assays quantified by flow cytometry or microscopy are the preferred methods to detect anti-MOG-type IgG in clinical settings. Fixed cell-based assays are a reasonable alternative, with assays considered clear positive with titres greater than or equal to 1:100 [1]. Immunosuppressants and corticosteroids are used as part of the treatment to prevent relapses and prevent further neurological damage [2].

Idiopathic intracranial hypertension (IIH) is a neurological condition that occurs when pressure increases within the skull without any apparent cause, including tumours or hydrocephalus. It mainly affects women of childbearing age, often with obesity, and manifests itself with symptoms such as intense headache, visual disturbances, and papilledema, which is the swelling of the optic nerve observed through fundoscopic examination. IIH rarely affects the paediatric population, being more common in children over 12 years old than in younger ones [4].

The diagnosis is made using clinical criteria, brain imaging to rule out other causes, and lumbar puncture to measure cerebrospinal fluid pressure. In patients with headaches, the diagnosis of IIH is made using the third version of the International Classification of Headache Disorders criteria. A cerebrospinal fluid (CSF) pressure that exceeds 250 mm (or 280 mm in obese children) is an essential criterion for a diagnosis of IIH [4]. Weight loss is part of the treatment, along with medications like carbonic anhydrase inhibitors and, in extreme cases, surgery to alleviate intracranial pressure [5].

We present the case of a boy with headache and brain MRI suggestive of idiopathic intracranial hypertension. This case was peculiar because a final diagnosis of MOGAD was made, although brain MRI never showed abnormalities typical of MOGAD. Furthermore, the patient did not respond to classic pharmacological treatments for IIH, but required steroid therapy.

## 2. Case Report

An 8-year-old boy was hospitalized for a headache that had been ongoing for three weeks. The headache was mainly frontal and involved throbbing pain that was not alleviated by acetaminophen or NSAIDs. The headache was daily, continuous, and non-remitting, but allowed night rest. Symptoms associated with headaches included photophobia, phonophobia, and vomiting, the latter occurring in the morning. There was no history of aura. There was no history of primary headache or other relevant health problems in the medical history. In particular, the boy had no fever and had not had any recent infections or trauma.

The general and neurological examinations were not remarkable. The optic disc showed bilateral swelling at the ocular fundus. The BMI of 23.5 was above the 95th centile for age. A 3 Tesla (T) brain magnetic resonance imaging (MRI) (including T1 and T2 weighted images, 3D FLAIR, axial DWI with reconstrued Apparent Diffusion Coefficient (ADC) maps without administration of contrast medium) showed a slight distension of the optic nerve sheaths, but all other cerebrovascular findings were normal. Blood tests were normal, including blood count; renal and liver function; thyroid hormones; vitamin C, A, and D dosage; celiac disease antibody dosage; C-reactive protein; cytomegalovirus; and Epstein–Barr virus antibodies.

The child underwent a lumbar puncture (LP), and the chemical–physical examination of the cerebrospinal fluid (CSF) revealed elevated protein (51 mg/dL) and white blood count (WB: 63 cell/UL). The cytological examination failed to detect any neoplastic cells, and the search for viruses and bacteria in the CSF was negative. Research assessing viruses and bacteria in the CSF were negative (polymerase chain reaction (PCR) test for Varicella Zoster, Herpes Simplex type 1 and 2, Cytomeg-alovirus, Epstein–Barr, and enterovirus and culture examination for mycobacteria are all negative).

IIH was suspected even though there was no CSF pressure measurement, and acetazolamide (750 mg/day) was used to treat it. Due to his headache becoming worse, he was transferred to our third-level neurology center. He underwent a PL, and his CSF pressure was measured at 75 cmH_2_O. The pressure was restored to normal values by removing CSF, which was analyzed, showing increased proteins (45 mg/dL) and cells (36 mm^3^). There were no abnormalities observed in the visual evoked potential (VEP) or electroretinogram (ERG). The headache disappeared within two days after the LP, and he was discharged from the hospital with the recommendation to continue taking acetazolamide.

**Figure 1:**
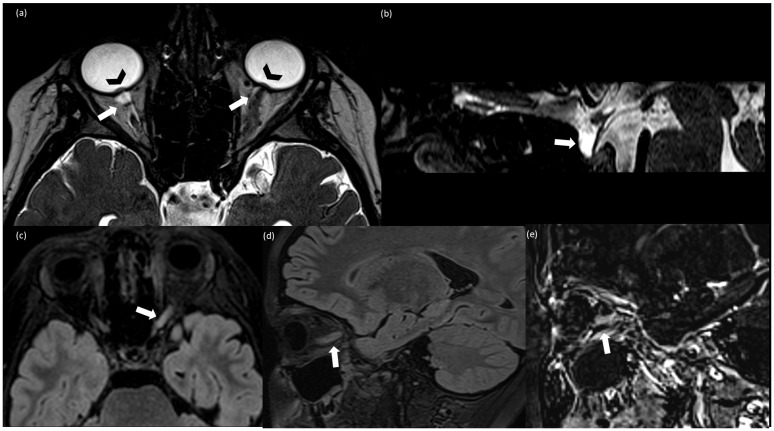
(**a**) Axial T2-weighted CISS (Constructive Interference Steady State); (**b**) sagittal T2-weighted CISS; (**c**) axial FLAIR (Fluid Attenuated Inversion Recovery; (**d**) sagittal T2 FLAIR; (**e**) sagittal subtraction images between post-contrast and pre-contrast T1 weighted MPRAGE. (**a**,**b**) Finding consistent with idiomatic intracranic hypertension (IIH): bilateral prominent subarachnoid space around the optic nerves (arrow), more evident on the right side because of the left optic nerve swelling, intraocular protrusion of the optic nerves (arrowhead) (**a**), and partially empty sella (arrow) (**b**); (**c**–**e**) findings consistent with optic neuritis (ON): T2-FLAIR hyperintensity and swelling of the left optic nerve (**c**,**d**) associated with contrast enhancement (**e**).

The child was hospitalized again after experiencing a sudden loss of vision in his left eye after four days. The eye exam revealed a visual impairment of 2/10 in the left eye (10/10 in the right eye) as well as a worsening of bilateral optic disc swelling. VEPs were absent in the left eye, but they were present in the right eye with normal latency and amplitude. A 3T brain and spinal cord MRI (performed at 3T, including T1 and T2 weighted images, 3D FLAIR, 3D T2-weighted CISS (Constructive Interference Steady State), axial DWI with ADC maps, and post-contrast T1 weighted images for the brain; sagittal T1 and T2 weighted images and post-contrast T1 weighted images for the spinal cord) showed findings suggestive of intracranial hypertension (IH), including bilateral prominent subarachnoid space around the optic nerves, intraocular protrusion of the optic nerves, flattening of the posterior globes, tortuous optic nerves, and partially empty sella (Figure 1a,b). Moreover, a T2-hyperintensity and swelling of the orbital and pre-chiasmatic tract of the left optic nerve was detected. There was a slight contrast enhancement of the left optic nerve compatible with optic neuritis (ON) (Figure 1c–e). There were no parenchymal abnormalities or spinal lesions. PL was repeated, and the pressure of the CSF was 70 cmH_2_O. We also found persistence of hyperproteinorrhachia (62 mg/dL) and leukocytosis (WBC 67 cell/UL). The pressure was brought back to normal by removing the CSF once more. The following blood tests were normal: blood count, liver and kidney functions, anti-nuclear (ANA), anti-neutrophilic granulocyte cytoplasm (ANCA), anti-phospholipid, anti-cardiolipin, anti-beta 2 glycoprotein1, anti-DNA and extractable nuclear antigen (ENA), anti SS-A/r, anti SS-B/la, and anti-aquaporin 4 (AQ4) antibodies.

Serum anti-MOG IgG was detected by fixed CBA at a dilution of 1:100. Anti-MOG IgG was also found in the CSF at a dilution of 1:100. End-point titration was not performed.

The CSF exhibits an oligoclonal pattern that is associated with the presence of at least three IgG-type bands. The simultaneous presence of ON and anti-MOG type IgG using cell-based assays allows for the diagnosis of MOGAD [1]

**Figure 2:**
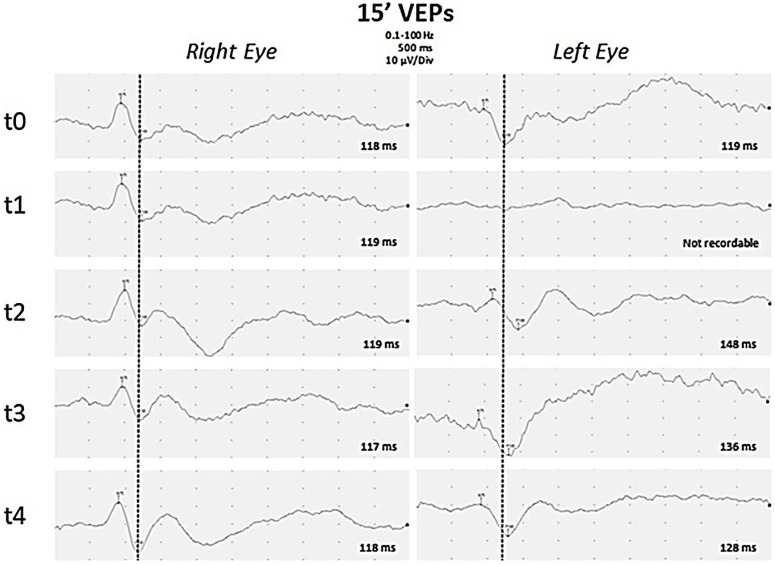
**Visual Evoked Potentials.** Black and white pattern reversal visual evoked potentials (PR-VEP) with a visual angle of 15 min of arc were recorded separately for each eye through silver–silver chloride cup electrodes placed over Oz and Cz (reference) with ground on the ear lobe. P100 latencies are reported for each PR-VEP. PR-VEPs performed at early onset of disease were bilaterally normal (t0). After one week (t1), P100 was absent on the left eye, but was still normal on the right eye. During follow-up, PR-VEPs showed gradual improvement on the left eye after eight days (t2), about one month (t3), and three months (t4) from the onset of disease. Finally a slight asymmetry of P100 latency was observed, increased by 10 ms on the left eye compared to the contralateral eye.

Intravenous (IV) methylprednisolone was started at a dose of 20 mg/kg/day for a 5-day period. Given the persistence of the visual deficit in the left eye, a course of plasmapheresis was proposed, but the parents refused this therapeutic option. Therefore, treatment with human immunoglobulin G was then performed intravenously at a dose of 2 g per kilogram per day in a single administration. The control VEPs performed 3 days after the end of IV therapy showed an improvement in the visual response, with absolute latency values of the P100, which were, however, confirmed to be altered in the left eye. The patient was discharged with the indication to continue prednisone (2 mg/kg/day), acetazolamide (750 mg/day), and topiramate (2 mg/kg/day). During the first follow-up visit one month later, the boy reported having no headache and better vision in his left eye. Fundus oculi showed improvement in the swelling of the optic disc. The gradual improvement of visive function (Figure 2) was revealed by VEP. The patient underwent another LP and discovered that the CSF pressure was at a normal level of 23 cmH_2_O.

After one month of treatment, he discontinued prednisone and topiramate and he remained on acetazolamide until the fundus oculi normalized three months after diagnosis of MOGAD. He continues to receive 2 g/kg of immunoglobulin IgG IV every month and is being advised to follow a low-calorie diet. He had no relapses with neurological symptoms during the 4 months follow-up. A neuroradiological check-up and anti-MOG dosage were scheduled for 6 months, but they have not been carried out yet as of the writing of this paper.

## 3. Discussion

The reported case is unique for the following three reasons: (1) It is possible that the headache and vomiting were symptoms of intracranial hypertension caused by MOGAD and not IIH; (2) the ON emerged only a month after the clinical onset; (3) MRI has never shown alterations in the brain or spine that are suggestive of MOGAD, but rather, alterations typical of IIH.

While there are various data in the literature that support how IH can occur in MOGAD during ADEM or ON episodes [2,3,4], there are few cases in which some symptoms of IIH, like headache and papilledema, have been reported as isolated symptoms in MOGAD [6,7,8,9,10]. Considering the ICHD-3 diagnostic criteria for IIH [8] and excluding ADEM cases, the literature on MOGAD with documented IH has reported three adult cases and six pediatric cases (Table 1) [6,7,8,9,11,12,13,14]. Among the pediatric cases, only 2/6 (n°5 and 6) have not had MRIs at onset showing alterations in the brain, spine, or optic nerve suggestive of MOGAD [7,8]. Alqahtani et al., 2023, reported a 12-year-old obese boy presenting with headache and bilateral asymmetric papilledema, raised intracranial pressure, and normal brain and spine imaging. In this case, the patient had a moderate CSF pressure of 35 cmH_2_O and responded to pharmacological treatment without recurrence of symptoms [7].

Valdrighi et al. described a case of a 12-year-old boy with IIH in whom the brain MRI during the acute phase of the symptoms was normal, while the follow-up after 4 months showed parenchymal lesions. However, it was not possible to establish with certainty when these alterations formed. The presence of leptomeningeal enhancement on an MRI during the onset of this case could suggest an inflammatory origin [8]. In our patient, the symptoms of IH and optic neuritis were separated by a time interval of around 1 month, and brain RMN remained negative for lesions typical of inflammatory demyelinating disease until the last follow-up (6 months).

In 5/6 patients, the presence of cells in the CSF [6,7,8,10,13] was documented, while hyperproteinorachia was documented in 2/6 [6,13]. The presence of pleocytosis could suggest HANDL (syndrome of headache accompanied with transient neurologic deficits and cerebrospinal fluid lymphocytosis), but the absence of transient neurological deficits at the onset excluded this diagnosis. Furthermore, on several occasions, the search for viruses, bacteria, or neoplastic cells was negative, excluding secondary causes of headache [10].

As in our case, three out of six patients were obese [7,8,9]. All patients received corticosteroids, and in one of six cases, chronic treatment with IgG iv was performed because of neuroradiological progression [8].

It is unlikely that there was a casual and non-causal link between MOGAD and IIH in our patients. The time interval between the onset of headache and optic neuritis was still short, and IH did not respond to therapies for IIH, but for MOGAD.

There is currently no evidence to prove the mechanism of IH in MOGAD. Narula et al. hypothesized that CSF flow dynamics and absorption were altered, partly because of proinflammatory factors [11]. In support of this thesis, it has been noted that, in cases of anti-MOG with isolated IH, white blood cell counts or proteins have been particularly elevated, reflecting a great degree of inflammation [6,7,8,10,13]. Also, the response to corticosteroids in these cases supports this theory [6,7,8,9,10,13].

It is interesting to note that obesity is linked to both IIH and inflammatory demyelinating diseases [4,11,15]. The association between obesity and IIH has long been recognized [4,16]. A recent systematic literature review explained how, among the possible mechanisms that contribute to raised intracranial pressure in obese children, there is reduced intracranial venous drainage as a result of increased intrathoracic and intra-abdominal pressures, which may lead to a decreased CSF absorption rate, eventually causing increased intracranial pressure [16]. An alternative hypothesis is that obesity causes a chronic proinflammatory state in the body, which may be strongly associated with the development of IIH [17]. There are multiple mechanisms that suggest that an inflammatory process plays a role in the pathogenesis of IIH, partly to explain the strong link between obesity and IIH. The enzyme 11β-hydroxysteroid dehydrogenase type 1 (11b-HSD1) may have a potential role in IIH by regulating CSF secretion [18]. It has been demonstrated that this enzyme is out of balance with increased activity in both obesity and IIH. In humans, 11b-HSD1 raises the level of local cortisol [19]. High cortisol levels for an extended period have been observed to enhance the production of pro-inflammatory mediators and, possibly, to enhance CSF production by interfering with sodium transporters in the choroid plexus. In IIH patients, 11b-HSD1-levels can be reduced by weight loss and lower ICP values [16,17,18,19]. The possible roles of proinflammatory cytokines and apokines such as leptin, IL-2, IL-10, IL-12, IL-17, and TNF-α have been studied in IIH. Increases or decreases in levels of these inflammatory mediators have been demonstrated in several studies [20,21,22], suggesting that pro-inflammatory activation could potentially be involved in the pathogenesis of IH [17].

Therefore, both obesity and MOGAD-related inflammation may be causes contributing to IIH development.

Recent research has revealed a connection between MOGAD and overweight, with a high BMI being reported more frequently than in individuals with multiple sclerosis and neuromyelitis optica spectrum disorder [23]. Especially in MOGAD patients with IH, a high frequency of obese patients has been reported (Table 1). Elevated BMI can be an important risk factor when diagnosing MOGAD and other demyelinating disorders in children [14,15]. Weight reduction therefore represents an important therapeutic strategy in these patients. However, it should be considered that in very young children, it can be difficult to have correct adherence to a low-calorie diet, and therefore, other lifestyle interventions (sports activity, adequate water intake) should also be encouraged.

## 4. Conclusions

The spectrum of MOGAD can include symptoms that can mimic IIH, and in this case, suspicious data could be derived from the finding of an increase in cells or proteins in the CSF analysis. In these cases, the dosage of anti-MOG should be considered.

Brain MRI may have a pivotal role, detecting findings of IIH, and the administration of contrast enhancement may help to evaluate an eventually associated ON.

Patients should be treated with steroids and acetazolamide, and in severe cases, excess CSF should be removed. Finally, long-term treatment with intravenous immunoglobulin or other immunosuppressants should be considered to prevent relapses.

## Figures and Tables

**Table 1:** Cases reported in the literature of patients with MOGAD with IIH-like onset. OCB: oligoclonal bands; VEP: visual evoked potentials; OCT: optical coherence tomography; NA not available; L: lymphocytes; N: neutrophils; M: monocytes; E: eosinophils.

Case/Reference	Age/Sex	Weight or BMI (kg/m^2^)	CSF Opening Pressure (cmH_2_O)	CSF Protein (mg/dL)	CSF White Blood Cell (count/mm^3^)	BOC	Associated MRI Findings to IHH	Clinical Findings	VEP/OCT	Treatment
**Adults Subjects**										
1/ Chaudhuri et al., 2022 [14]	19/F	NA	35	Normal	20 (lymphocytes)	No	Leptomeningeal enhancement	Blurred vision, severe headache, vomiting	NA	Methylprednisolone, mycophenolate, rituximab
2/ Jeantin et al., 2022 [12]	35/F	NA	31	NA	189 (lymphocytes)	Yes	Unilateral ON, brain and spinal lesions	Headaches, nausea, abdominal pain, confusion	NA	Acetazolamide, methylprednisolone, azathioprine
3/ Lotan et al., 2018 [10]	40/M	32	33	Normal	Normal	NA	Bilateral ON	Blurred vision, headache	NA	Acetazolamide, methylprednisolone
**Pediatric Subjects**										
4/ Maran et al., 2023 [9]	13/M	44.8	65	NA	NA	NA	Intrasellar arachnoid herniation; T2 hyperintense subcortical lesions in the brain	Headache, nausea, vomiting, right eye sudden vision loss	OCT: progressive thinning in temporal and inferotemporal sectors and bilateral papilledema	Acetazolamide Methylprednisolone Neurosurgical treatment of IH.
5/ Alqahtani et al., 2023 [7]	12/M	28.8	35	Normal	75 (89% L, 4% N, 3% M, 1% E)	No	flattening of the posterior globes, prominent both Meckel cave, optic nerves tortuosity	Headache Bilateral reduced visual acuity	asymmetric bilateral papilledema and abnormal visual acuity and visual field.	Acetazolamide, methylprednisolone 30 mg/kg/day was administered for 5 days followed by low doses acetazolamide 10 mg/kg/day (duration treatment 3 months)
6/ Valdrighi et al., 2021 [8]	12/M	BMI not specified Obese	52	Normal	157 (68% L, 11% N, 17% M, 4% E)	No	a partially empty sella, intraocular protrusion of the optic nerves, flattening of the posterior globes, narrowing of the venous sinuses, and tortuous optic nerves multifocal leptomeningeal enhancement. After 4 months from the onset: T2 hyperintense cerebellar and pontis lesions	Blurred vision, nausea, vomiting, headaches, cranial nerve VI palsies.	Bilateral papilledema Normal visual acuity and visual field.	Acetazolamide, methylprednisolone. After radiological relapse: monthly IV immunoglobulin
7/ Narayan et al., 2019 [13]	18/F	NA	29	48	57–82 (84–97% L)	No	bilateral ON and multiple spinal cord lesions	Bilateral blurred vision, headache, photophobia	Bilateral papilledema and poor visual acuity	Five cycles of plasma exchange Acetazolamide and Methylprednisolone (duration 1 month)
8/ Lotan et al., 2018 [10]	6/F	18	35	Normal	8	NA	Brain and spinal lesions	Right eye reduced vision, headache	Bilateral papilledema	Acetazolamide, methylprednisolone
9/Zhou et al., 2023 [6]	12/M	NA	>36	158	252		Multifocal cortical/subcortical T2 hyperintensities, bilateral ON head elevation, and enlarged, T2 hyperintense intraorbital optic nerves	Bilateral blurry vision, facial droop, slurred speech, and ataxia	Poor visual acuity papilledema	Methylprednisolone iv,

## Data Availability

The original contributions presented in the study are included in the article. Further inquiries can be directed to the corresponding author.
